# Effects of distributed leadership on teachers’ job satisfaction: The mediating role of teacher collaboration

**DOI:** 10.1371/journal.pone.0347862

**Published:** 2026-04-30

**Authors:** Li Ma, Ying Gu

**Affiliations:** 1 College of Teachers, Chengdu University, Chengdu, China; 2 Chengdu Jinxi Foreign Language Experimental Primary School, Chengdu, China; Grigore T Popa University of Medicine and Pharmacy Iasi: Universitatea de Medicina si Farmacie Grigore T Popa lasi, ROMANIA

## Abstract

Teachers are the primary resource for educational development and the fundamental force behind building and achieving a high-quality education system. Researching strategies to improve teacher job satisfaction is crucial for the high-quality development of teachers. Being empirical in nature and based on distributed leadership theory, this study uses data from OECD’s TALIS 2018 teachers survey. The multiple regression analysis model is used to analyze the impact of distributed leadership on teachers’ job satisfaction in an international context, with a particular focus on the mediating effect of teacher collaboration. Results show that distributed leadership significantly and positively predicts teachers’ job satisfaction, and teacher collaboration partially mediates the relationship between distributed leadership and teacher job satisfaction. The study not only provides a systematic explanation and empirical evidence for the promotion and enhancement mechanisms of distributed leadership on teachers’ job satisfaction from an international perspective, but also offers valuable insights for finding pathways to improve teachers’ job satisfaction and promote teacher development.

## Introduction

Teachers are the first resource for educational development and the fundamental force for building a high-quality education system and achieving high-quality education. The 2030 Agenda for Sustainable Development of the United Nations Educational, Scientific and Cultural Organization (UNESCO) establishes the sustainable development goal for education as “Sustainable Development Goal 4 (SDG 4): Ensure inclusive and equitable quality education and lifelong learning opportunities for all” [1].

The “Education 2030 Framework for Action” provides guidance for implementing this grand goal and commitment. The 2030 Framework proposes “ensuring an inclusive and equitable quality education system for all”, and emphasizes that “teachers are at the core of providing quality education and play a key role in achieving Sustainable Development Goal 4”. Teachers are crucial to ensuring the quality of education, and building a high-quality teaching workforce is essential for providing equitable and quality education [2]. In recent years, many countries around the world have actively promoted the construction of high-quality teacher teams and have achieved certain results. However, the increasing challenges and pressures faced by teachers in their current work, including increased work pressure, higher workloads and increased uncertainty in the teaching and learning environment [3], have contributed to the generally low level of job satisfaction among teachers [4]. Coupled with a particularly acute shortage of teachers [5], the stability and development of the teaching force is greatly challenged. The Global Teacher Report-Teacher Shortages states that “teacher shortages will have far-reaching consequences in terms of reduced teacher well-being” [5]. Teachers’ job satisfaction is not only related to their work status, career mobility, burnout and psychological well-being [6,7], but also directly affects students’ academic performance and physical and mental health [8]. Therefore, analyzing the working conditions of teachers and exploring strategies to enhance teachers’ job satisfaction is particularly important for solving teacher recruitment, attractiveness, and retention issues [5].

Exploring the dependent variables and influence mechanisms affecting teachers’ job satisfaction will provide empirical evidence for exploring effective ways to enhance teachers’ job satisfaction. Research has confirmed that teachers’ job satisfaction can be affected by teachers themselves [9], school organizations [10] and social environments, among which the relationship between distributed leadership and teachers’ job satisfaction has been effectively verified in western empirical studies [11]. “How does distributed leadership affect teachers’ job satisfaction? “ has become a focus of attention in today’s education field. In view of this, based on the survey data of the Organization for Economic Cooperation and Development (OECD), we explore the impact of distributed leadership on teachers’ job satisfaction, and further reveals the mechanism of distributed leadership’s role in influencing teachers’ job satisfaction in the international context from the perspective of teacher collaboration.

This study makes several contributions to the literature. First, it extends distributed leadership theory by empirically testing its effects on teachers’ job satisfaction in an international context, moving beyond the predominantly Western, single-country focus of prior research. Second, it unpacks the ‘black box’ of how distributed leadership influences job satisfaction by identifying teacher collaboration as a significant mediating mechanism—a relationship that has been theoretically suggested but rarely tested with large-scale cross-national data. Third, by leveraging the OECD TALIS 2018 dataset, the study provides robust, generalizable evidence that informs policy discussions on teacher retention and school improvement from a distributed leadership perspective.

## Literature review

### Distributed leadership and teachers’ job satisfaction: Interrelationship

Job satisfaction has been a hot topic of research since the 1930s, due to the fact that job satisfaction is an important predictor of employee performance [12] and employee retention [13,14]. Although there is considerable research on job satisfaction, there is no consensus on its definition. Existing research suggests that job satisfaction can be measured on both affective [15] and cognitive [16,17] dimensions. From an affective perspective, job satisfaction is “a pleasant or positive emotional state resulting from an individual’s appraisal of a job or work experience” [18]; from a cognitive perspective, job satisfaction is the difference between an individual’s perceived or expected level of a job [19]. Due to the complexity of the nature of the teaching profession, teachers’ job satisfaction is a multidimensional structure [20] that is influenced by both internal individual factors and external environmental factors: first, from the teacher’s own level, teachers’ perceived sense of efficacy, occupational stress and burnout, teachers’ workloads, teachers’ remuneration packages, and teachers’ professional development [21] affect teachers’ job satisfaction; second, from the external school level, the organization and culture of the school have a significant impact on teachers’ job satisfaction. The school working environment is one of the key factors in teachers’ job satisfaction. A favorable working environment can stimulate teacher enthusiasm for work and improve their efficiency, thus increasing job satisfaction; the cooperative atmosphere of the school also has an impact on teachers’ job satisfaction. A cohesive and congenial cooperative atmosphere where teachers can support, collaborate, and grow together is conducive to job satisfaction; in addition, group interpersonal relationships are an important factor influencing teachers’ job satisfaction [22]; and school leadership is a key predictor of teacher career satisfaction [23]. Both distributed leadership and instructional leadership have a positive direct impact on teachers’ job satisfaction [24].

Distributed leadership can be traced back as far as 1954 by Cecil Gibb [25], while distributed leadership as a theory of educational leadership began in the 1990s. Different scholars have interpreted the concept of distributed leadership in different ways. Mayrowetz understands distributed leadership in four ways: (1) a theoretical framework for educational leadership; (2) a style of leadership that fosters democracy in schools; (3) a method for improving school outcomes (e.g., efficiency and effectiveness); and (4) a style of leadership used to enhance professional learning [26]. According to Harris, both formal leaders in administrative positions, subject leaders, key teachers, etc., play the role of leaders in distributed leadership. These leaders are not just people who hold specific positions, but also influence teachers and students at all levels of the school [27]. Spillane et al. define distributed leadership as the practice of shared decision-making at multiple levels [28], a definition that has been widely accepted in academia.

Distributed leadership is a school leadership theory that is now widely used internationally [29,30] and is based on the concepts of empowerment, collaboration and sharing [31], with an emphasis on the shared leadership functions of headmasters, teachers and other organizational members [32]. In recent years, there has been a rapid increase in research on distributed leadership in schools, and scholars have gradually included teachers’ job satisfaction in their studies to explore the relationship between distributed leadership and teachers’ job satisfaction. The study found that when teachers had the opportunity to participate in decision-making and were empowered, they demonstrated higher levels of job satisfaction [33]. In addition, distributed leadership was positively and directly related to teachers’ job satisfaction [24]. Using Singaporean teachers as example, Torres found that distributed leadership had a significant effect on teachers’ job satisfaction; higher distributed leadership scores were associated with higher teachers’ job satisfaction scores [34]. Based on the Chinese cultural context, it was found that distributed leadership has a significant positive impact on teachers’ job satisfaction [35], and it was found that the implementation of distributed leadership is conducive to enhancing teachers’ job satisfaction. In addition, schools can stimulate teacher self-efficacy [24] by empowering them [36] and shaping a supportive school culture to enhance their job satisfaction.

### The mediating role of teacher collaboration

Teacher collaboration is an interactive process involving two or more team members developing a plan or solving a problem [37], which requires teachers to share information, make decisions together, and work together with the aim of strengthening interdependent relationships among teachers [38]. Teacher collaboration is a necessary condition for educational reform [39], and it has a positive educational impact on students, teachers, and schools. Specifically, for students, teacher collaboration is beneficial for improving students’ understanding and performance [40]. For teachers, collaboration can reduce feelings of isolation, enhance teacher self-efficacy, and increase motivation, as well as reduce teacher workload and improve efficiency [41–43]. For schools, teacher collaboration is conducive to creating an innovative atmosphere [44] and forming a flatter power structure [45]. In educational activities, teacher collaboration does not always achieve positive results, so it is necessary to consider the potential risks, namely when teachers lose autonomy [46] and take on more workload [47].

Teacher collaboration has been found to be a significant factor in teachers’ job satisfaction [48,49] and it significantly predicts teachers’ job satisfaction [48]. Teacher collaborative practices, which include collaborative activities or learning communities for teaching and learning, help teachers to share their experiences and knowledge within the school organization, which in turn leads to higher levels of satisfaction exhibited by teachers [50]. There was also a strong link between headmaster leadership and teacher collaboration [51], with different headmaster leadership types having different effects on teacher collaboration, with administrative authoritative leadership being negatively related to teacher collaboration [51] and distributed leadership significantly and positively predicting teacher collaboration [52,53].

Based on structural equation modelling, it was found that teacher collaboration was a mediating variable between distributed leadership and teachers’ job satisfaction [20], and that distributed leadership significantly influenced teachers’ job satisfaction through teacher collaboration [35]. The implementation of distributed leadership in schools can promote collaboration and sharing among teachers, thereby further enhancing their job satisfaction [54].

Based on the comprehensive theoretical and empirical review, the following hypotheses are proposed:

**H1:** Distributed leadership positively predicts teachers’ job satisfaction.

**H2:** Teacher collaboration mediates the relationship between distributed leadership and teachers’ job satisfaction.

**Fig 1:**
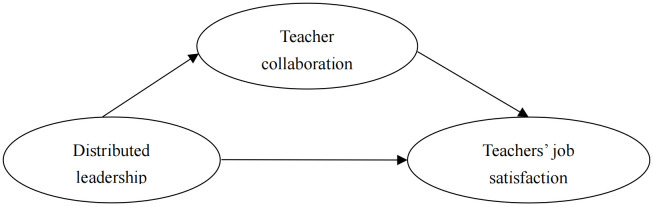
Hypothetical model diagram.

The hypothesized mediation model is presented in Fig 1.

## Research methodology

This section describes the data source, sample characteristics, measurement of key variables, and the analytical methods used in this study.

### Data sources and sample

The data used in this study are derived from the Teaching and Learning International Survey (TALIS) 2018. TALIS 2018 is an international survey conducted by the OECD, which aims to understand the national/regional teachers’ classroom teaching, their professional development and other actual situation and provide useful information for education policy makers and education researchers. In this study, based on the consideration of variables such as teachers’ personal characteristics (e.g., gender, academic qualifications, etc.), school characteristics (e.g., geographic location, number of students, etc.), and teacher collaboration, the teachers’ and headmasters’ datasets were merged, and data responsiveness screening was conducted on the entire initial sample to eliminate invalid data. After screening, this study ultimately obtained a valid sample of 197,018 teachers from OECD countries. Among them, 68.57% were female teachers, numbering 135,086, and 31.43% were male teachers, totaling 61,932. In terms of educational qualifications, teachers with less than a bachelor’s degree accounted for 6.47%, totaling 12,744; teachers with a bachelor’s degree accounted for 55.49%, totaling 109,322; and teachers with a postgraduate degree accounted for 38.05%, totaling 74,952.

### Variable measures

The variable measures in this study included distributed leadership, teacher collaboration, and teachers’ job satisfaction, while strictly controlling for other variables that may affect teachers’ job satisfaction.

### Distributed leadership

For the distributed leadership variable, TALIS 2018 included three items, such as “Schools give teachers the opportunity to actively participate in school decision-making”. All three items were scored on a four-point scale, with all questions scored from 1 (*strongly disagree*) to 4 (*strongly agree*), with higher scores indicating higher levels of distributed leadership. The Cronbach’s alpha coefficient of the scale was 0.83 through the reliability test, the average of the three items was used as the score for this variable.

### Teacher collaboration

Regarding the teacher collaboration variable, TALIS 2018 includes eight items on professional collaboration, such as “Participation in professional learning collaborations”. All eight items were scored using a six-point scale, with options based on the frequency of the behavior as follows: “Never”, “Once a year”, “2-4 times a year”, “5-10 times a year”, “1-3 times a month”, and “once a week or more”, corresponding to scores of 1–6, respectively. Higher scores indicate more frequent collaborative activities among teachers within the school. Reliability testing yielded ta Cronbach’s alpha coefficient of 0.79 for this scale, and the average of the eight items was used as the score for this variable.

### Teachers’ job satisfaction

With regard to the teachers’ job satisfaction variable, TALIS 2018 includes seven items, such as “Working at this school makes me physically and mentally happy”. All seven items are rated on a four-point scale, with options ranging from 1 (*strongly disagree*) to 4 (*strongly agree*). Higher scores indicate higher job satisfaction among teachers. The reliability test showed that the Cronbach’s alpha coefficient of the scale was 0.78. In this study, the seven items of teachers’ job satisfaction were analyzed in detail and synthesized on the basis of them to obtain an overall score of teachers’ job satisfaction. A composite score was created by averaging all seven items, providing an overall indicator of teachers’ job satisfaction.

### Control variables

In order to fully examine the mechanism of distributed leadership’s influence on teachers’ job satisfaction, we strictly controlled for a variety of variables that may affect teachers’ job satisfaction in the empirical analyses, such as gender, educational attainment, years of teaching experience, professional certification status, school type (public vs. private), and school location. The inclusion of these control variables helps to isolate the unique effect of distributed leadership on teachers’ job satisfaction.

### Data analysis methods

The data analyzed using SPSS 22.0 and Stata 16.0. First, we performed reliability tests, descriptive statistics, and correlation analysis. Secondly, we explored the impact of distributed leadership on teachers’ job satisfaction through multiple regression analysis. Finally, the bootstrap method was used to test the mediating effect of teacher collaboration, with a sampling number of 1,000, and the significance of the mediating effect was judged based on whether the bias-corrected 95% confidence interval contained zero, in order to further examine the mechanism through which distributed leadership affects teachers’ job satisfaction.

## Results of data analysis

### Descriptive statistics and correlation tests for variables

Descriptive statistics and bivariate correlations for all variables are presented in Table 1. The correlation matrix reveals that distributed leadership demonstrated a significant positive correlation with teachers’ job satisfaction (r = 0.337, *p <* 0.001) and with teacher collaboration (r = 0.223, *p <* 0.001). Simultaneously, teacher collaboration was positively correlated with job satisfaction (r = 0.225, *p <* 0.001). This pattern of interrelationships among the three core variables aligns with theoretical expectations and provides preliminary support for testing the proposed mediation model.

**Table 1:** Means, standard deviations and correlation coefficients of variables.

Var	M	SD	1	2	3	4	5	6	7	8
Sat	2.967	0.493	1							
Dis	2.855	0.601	0.337***	1						
Coo	3.494	1.008	0.225***	0.223***	1					
Gen	1.314	0.465	−0.007***	−0.022***	−0.061***	1				
Edu	5.295	0.721	−0.053***	0.012***	−0.079***	−0.042***	1			
Teat	9.632	8.958	0.008***	0.030***	−0.029***	−0.045***	0.043***	1		
Loc	3.036	1.335	0.006***	−0.002	0.008***	0.007***	0.001	−0.011***	1	
Pub	1.210	0.408	0.021***	−0.006***	0.033***	−0.003	−0.012***	−0.021***	0.265***	1

Note: + *p <* 0.1; *p < 0.05; **p < 0.01; ***p < 0.001.

Regarding control variables, gender showed a weak but significant negative correlation with teachers’ job satisfaction (r = −0.007, *p <* 0.001), while teaching experience exhibited a slight positive correlation with job satisfaction (r = 0.008, *p <* 0.001). School location demonstrated a weak correlation with job satisfaction (r = 0.006, *p <* 0.001), and school type (public/private) was positively but weakly associated with job satisfaction (r = 0.021, *p <* 0.001). Although statistically significant, the generally small magnitude of these correlations between control variables and the main research variables suggests that while they need to be controlled for in subsequent regression analyses, they are unlikely to alter the fundamental relationships among the core variables.

### Hypothesis testing

#### Direct effects.

The results of the regression analyses are presented in Table 2. Model 1, 2, and 4 specified teachers’ job satisfaction as the dependent variable, while Model 3 examined teacher collaboration as the dependent variable. After controlling for extraneous variables, distributed leadership demonstrated a significant positive effect on teachers’ job satisfaction (β = 0.28, *p <* 0.001) in Model 1, indicating that teachers who perceive greater opportunities to participate in school decision-making report higher levels of job satisfaction. This finding supports Hypothesis H1.

**Table 2:** Results of stratified regression analyses.

Var	Model 1	Model 2	Model 3	Model 4
	Sat	Coo	Sat	Sat
dis	0.280*** (0.002)	0.378*** (0.004)		0.252*** (0.002)
Coo			0.108***	0.073***
Gen	−0.001 (0.002)	−0.124*** (0.005)	0.007** (0.002)	0.008*** (0.002)
Edu	−0.040*** (0.001)	−0.117*** (0.003)	−0.026*** (0.002)	−0.031*** (0.001)
Teat	0.000 (0.000)	−0.004*** (0.000)	0.001*** (0.000)	0.000*** (0.000)
Loc	0.001 (0.001)	−0.001 (0.002)	0.001 (0.001)	0.001^+^ (0.001)
Pub	0.021*** (0.003)	0.068*** (0.006)	0.013*** (0.003)	0.016*** (0.003)
_cons	2.349***	3.157***	2.688***	2.117***
N	197018	197018	197018	197018
r2_a	0.120	0.063	0.051	0.141

Note: + *p <* 0.1; *p < 0.05; **p < 0.01; ***p < 0.001.

To examine the mediating mechanism, we followed the analytical procedure proposed by Baron and Kenny. Model 2 revealed that distributed leadership significantly and positively influenced teacher collaboration (β = 0.378, *p <* 0.001), suggesting that distributed leadership fosters a more collaborative environment among teachers. Meanwhile, Model 3 indicated that the mediating variable, teacher collaboration, exerted a significant positive effect on teachers’ job satisfaction (β = 0.108, *p <* 0.001), confirming that collaborative activities themselves contribute to teachers’ satisfaction with their work.

To further validate the potential indirect effect of distributed leadership on teachers’ job satisfaction, we included distributed leadership and teacher collaboration together in the regression analysis model. The results of the cascade regression analysis are shown in Model 4, where the coefficient c’ = 0.252 (*p <* 0.001), the coefficient b = 0.073 (*p <* 0.001), and the adjusted R2 is 0.141. The reduction in the coefficient for distributed leadership from Model 1 (β = 0.28) to Model 4 (β = 0.252), combined with the significant effect of teacher collaboration, provides initial evidence of partial mediation. Thus, the results from Models 1–4 collectively indicate that teacher collaboration partially mediates the relationship between distributed leadership and teacher satisfaction, supporting Hypothesis H2.

#### Mediation effect test.

In order to test the mediating effect of teacher collaboration, we employed bootstrap analysis with 1,000 resamples, judging significance based on whether the bias-corrected 95% confidence interval contained zero. This approach is preferred over the causal steps approach as it does not assume normality of the sampling distribution and provides a direct test of the indirect effect. First, constructing Model 1 to represent distributed leadership affecting teachers’ job satisfaction through teacher collaboration. Second, constructing Model 2 to represent the direct impact of distributed leadership on teachers’ job satisfaction. Third, constructing Model 4 to represent the mediating role of teacher collaboration between distributed leadership and teachers’ job satisfaction.

As shown in Table 3, bs1 represents the mediating effect, which is the product of a and b, and bs2 represents the direct effect. The mediation effect value for the path “distributed leadership→teacher collaboration→teachers’ job satisfaction” is 0.028, with a 95% confidence interval [0.027, 0.029] that does not include zero, confirming that the indirect effect is statistically significant. The direct effect remains significant (c’ = 0.252, *p <* 0.001), indicating partial mediation.

**Table 3:** Mediation test results for Bootstrap teacher collaboration.

	Observed Coef.	Bootstrap Std. Err.	z	P	95% CI
bs1r(indff)	0.028	0.001	54.69	< 0.001	[0.027, 0.029]
bs2r(dirff)	0.252	0.002	123.30	< 0.001	[0.250, 0.256]

## Discussion and conclusions

This study investigated the relationship between distributed leadership and teachers’ job satisfaction, with a particular focus on the mediating role of teacher collaboration. Drawing on data from 197,018 teachers across OECD countries in TALIS 2018, the analysis yielded three key findings, which we discuss below in relation to the existing literature.

First, distributed leadership significantly and positively predicts teachers’ job satisfaction. This result is consistent with the findings of scholars such as Torres [20]and Hulpia [55], further confirming Hypothesis H1. As a contemporary model of school management, distributed leadership emphasizes the sharing of power and responsibility between organizational members (e.g., headmasters and teachers) and helps to stimulate diverse teacher participation and collaborative leadership. This model fosters teamwork and encourages teachers to be actively involved in school affairs and to share responsibility, thus creating a more coordinated and efficient leadership mechanism. Under this leadership model, each teacher has the opportunity to contribute their expertise, while also gaining greater autonomy, a stronger sense of responsibility and belonging, and access to a wider range of resources. As a result, teachers develop a more positive attitude toward work and a stronger sense of well-being, which in turn contributes to school effectiveness. Given the importance of teachers’ job satisfaction to the high-quality development of schools, the school governance model should be deepened and transformed to enhance teachers’ job satisfaction. As the effectiveness of distributed leadership is gradually verified, it provides effective theoretical references and practical guidance for improving teachers’ job satisfaction.

Second, distributed leadership significantly and positively affects teacher collaboration (β = 0.378, *p <* 0.001). This finding suggests that the distributed leadership is effective in increasing the level of trust and collaboration among teachers. Distributed leadership emphasizes the decentralization and sharing of leadership. This type of leadership can create a fair and just working environment for teachers, enabling them to have more autonomy and participation in the teaching and learning process, as well as providing them with abundant opportunities for training and further education. These conditions stimulate their motivation and spirit of cooperation [56], and thus facilitating communication and collaboration among them [57]. Distributed leadership can provide teachers with a more relaxed space for development [58] and help them better cope with the challenges in education and teaching.

Third, teacher collaboration partially mediates the relationship between distributed leadership and job satisfaction. This finding aligns with prior research [20,24] and confirms our hypothesis H2. The mediation effect suggests that distributed leadership empowers teachers, fostering a more proactive and cooperative environment. This positive and collaborative atmosphere, in turn, enhances collective job satisfaction. Consistent with our findings, a recent study by Fan et al. [59] using TALIS 2018 Shanghai data also confirmed that distributed leadership positively predicts teacher job satisfaction, further underscoring the cross-cultural relevance of this relationship. Taken together, these findings theoretically elucidate the mechanism through which distributed leadership improves job satisfaction and offer valuable insights for school leadership practice.

### Recommendations for countermeasures

First, we recommended enhancing distributed and teacher leadership to realize its full potential within schools. The complexity of modern schools necessitates moving beyond a model where power is concentrated solely in the headmaster. Instead, leadership responsibilities should be shared and delegated to team members based on their expertise, fostering co-governance. Therefore, future training should focus on two areas: [60] First, to develop and strengthen headmasters’ sense of decentralization. Optimizing the content of headmaster training to enhance headmasters’ sense of decentralization, so that they actively assign leadership functions when faced with different school tasks and provide strong support to teachers in the form of performance responsibility incentives. Second, attention is paid to and the level of teacher leadership capacity is enhanced. The assumption of leadership roles by teachers is conducive to the enhancement of teacher commitment to their work and the all-round development of distributed leadership at all levels in the school, thereby contributing to the improvement of the overall quality of education in the school.

Second, to fully realize the positive impact of distributed leadership on teachers’ job satisfaction, its implementation should explicitly address the mediating role of teacher collaboration. While teacher collaboration is fostered by distributed leadership, it independently contributes to job satisfaction. Common collaborative activities—such as joint lesson planning, teaching-research groups, academic exchanges, interdisciplinary projects, and mentorship—constitute vital sources of professional fulfillment. Based on our findings, we recommend promoting teacher collaboration through two complementary approaches: intrinsic value construction and external support.

First, schools should actively communicate the value of collaboration, helping teachers appreciate its significance and cultivating their willingness to engage proactively. Second, systematic external support must be provided to strengthen teachers’ collaborative competence. This includes professional development—such as theoretical training that deepens understanding of collaboration’s meaning and practical applications—as well as structural support through guided practice, constructive feedback, and ongoing evaluation. Concrete measures may include establishing professional learning communities, organizing regular teaching-research activities, and creating platforms for experience sharing. In summary, advancing teacher collaboration through these multifaceted efforts will help amplify the beneficial effect of distributed leadership on teachers’ job satisfaction.

Third, educational leaders should adapt their leadership strategies to specific contexts to maximize the effect of distributed leadership on teachers’ job satisfaction. While distributed leadership inherently involves sharing responsibility and decision-making across the team, its effective implementation requires leaders to remain attentive to teachers’ individual needs and characteristics. By developing context-sensitive approaches, leaders can tailor their support to different school phases, tasks, and settings. For instance, more flexible and innovative strategies may be needed to stimulate teacher motivation and creativity when schools face new challenges, whereas periods of stable development may call for greater emphasis on coordination and integration across departments and teaching teams.

To formulate such personalized leadership plans, leaders should cultivate a thorough understanding of teachers’ professional needs and provide targeted support accordingly. This includes offering abundant professional development and training opportunities, as well as fostering a fair and supportive working environment. Through such adaptive leadership, the implementation of distributed leadership can be strengthened, thereby further enhancing teachers’ job satisfaction. This approach is exemplified in Nordic countries such as Finland and Sweden, where educational leaders prioritize teacher well-being and satisfaction by providing favorable working conditions and psychological support, which in turn boosts motivation and educational quality.

### Limitations and prospects

This study has several limitations that also represent promising avenues for future research. First, while the use of TALIS 2018 data offers broad international coverage, the findings are constrained by the lack of cross-national comparative analysis. Future studies could employ multigroup structural equation modeling (SEM) to systematically examine whether the mediation model tested in this study functions differently across cultural or policy contexts. Such comparative work could uncover important boundary conditions and yield richer insights into how national education systems shape the exercise and outcomes of distributed leadership.

Second, the cross-sectional nature of the data limits causal inference regarding the relationships among distributed leadership, teacher collaboration, and job satisfaction. Longitudinal or experimental designs are needed to trace how these variables influence one another over time and to establish stronger causal claims. A longitudinal design would be particularly valuable for capturing the dynamic processes through which leadership practices evolve and collectively influence teacher attitudes and school climate.
